# The Change from Past to Future for Adsorbent Materials in Treatment of Dyeing Wastewaters

**DOI:** 10.3390/ma6115131

**Published:** 2013-11-08

**Authors:** George Z. Kyzas, Jie Fu, Kostas A. Matis

**Affiliations:** 1 Laboratory of General & Inorganic Chemical Technology, Department of Chemistry, Aristotle University of Thessaloniki, Thessaloniki GR 541 24, Greece; E-Mail: kamatis@chem.auth.gr; 2 Department of Petroleum and Natural Gas Technology, Technological Educational Institute of Kavala, Kavala GR 654 04, Greece; 3 Environmental Engineering Program, Department of Civil Engineering, Auburn University, Auburn, AL 36849, USA; E-Mail: jzf0017@auburn.edu

**Keywords:** adsorption, dyeing wastewater, sustainability, past, future

## Abstract

Adsorption is one of the most promising decolorization techniques in dyeing wastewater treatment. Adsorption techniques for wastewater treatment have become more popular in recent years owing to their efficiency in the removal of pollutants too stable for biological methods. Dye adsorption is a result of two mechanisms (adsorption and ion exchange) and is influenced by many factors as dye/adsorbent interaction, adsorbent’s surface area, particle size, temperature, pH, and contact time. The main advantage of adsorption recently became the use of low-cost materials, which reduces the procedure cost. The present review firstly introduced the technology process, research history and research hotspot of adsorption. Then, the application of adsorption in treatment of dyeing wastewaters in the past decades was summarized, revealing the impressive changes in modes, trends, and conditions. From this review article, the different philosophy of synthesis of adsorbent materials became evident.

## 1. Introduction

A first obvious change from past to present, where the things began to change, was the discovery of synthetic dyes. Cheaper to produce, brighter, more colors—fast and easy to apply to fabric are some of the characteristic of those new dyes. Scientists have competed to formulate gorgeous new colors, and synthetic dyes had become obsolete for most applications. No doubt, this bright colored material has changed the world; however, the chemicals used to produce dyes are often toxic, carcinogenic, or even explosive [1]. Among the different pollutants of aquatic ecosystem, dyes are a major group of chemicals [2,3,4,5]. Many industries like textiles, leather, cosmetics, paper, printing, plastics, *etc.*, use many synthetic dyes to color their products. Thus, effluents from these industries contain various kinds of synthetic dyestuffs. For instance dyes used in the textile industries are classified into three classes: (a) anionic (direct, acid, and reactive dyes); (b) cationic (all basic dyes); and (c) non-ionic (dispersed dyes). Basic and reactive dyes are extensively used in the textile industry because of their favorable characteristics of bright color, being easily water soluble, cheaper to produce, and easier to apply to fabric [6,7,8].

Over 100,000 commercially available dyes exist and more than 700,000 t per year are produced annually [9,10]. Due to their good solubility, synthetic dyes are common water pollutants and they may frequently be found in trace quantities in industrial wastewater. An indication of the scale of the problem is given by the fact that two per cent of dyes that are produced are discharged directly in aqueous effluent [9,11]. Due to increasingly stringent restrictions on the organic content of industrial effluents, it is necessary to eliminate dyes from wastewater before it is discharged. Many of these dyes are also toxic, and even carcinogenic, and this poses a serious hazard to aquatic living organisms [12,13]. However, wastewater containing dyes is very difficult to treat, as the dyes are recalcitrant organic molecules, resistant to aerobic digestion, and are stable to light, heat, and oxidizing agents [14,15].

The most studied dye classes, in the dye bearing effluent treatment, are reactive and basic [16,17,18]. The dye loss from the dyeing process to the effluent is estimated 10%–50% for reactive dyes and 0–5% for basic ones [19]. Given that reactive and basic dyes could simultaneously exist in the equalization tank of a dye-house, it is of fundamental importance to remove both of them [20]. The research on dyeing wastewater treatment has been often focused on reactive dyes for three main reasons: (i) reactive dyes represent an increasing market share, because they are used to dye cotton fibers, which makes up about half of the world’s fiber consumption; (ii) a large fraction, typically around 30% of the applied reactive dyes, is wasted due to the dye hydrolysis in alkaline dye bath; and (iii) conventional wastewater treatment plants have a low removal efficiency for reactive and other anionic soluble dyes, which leads to colored waterways [17,21].

The presence of color and color-causing compounds has always been undesirable in water for any use. It is, therefore, not at all surprising to note that the color in wastewater has now been considered as a pollutant that needs to be treated before discharge. Thus, color removal is one of the most difficult requirements to be faced by the textile finishing, dye manufacturing, pulp and paper industries, among others. These industries are major water consumers and are, therefore, a source of considerable pollution. In order to implement an appropriate treatment process, it is of utmost importance to minimize pollution, and to do that, it is necessary to know its exact nature. Robinson *et al.* [11] attempted to give some collective information related to current available technologies and have suggested an effective, cheaper alternative for dye removal and decolorization applicable on large scale. They have also provided some important data related to the desorption of individual textile dyes and a synthetic dye effluent from dye-adsorbed agricultural residues using solvents [22,23], which is also important.

Adsorption technique for wastewater treatment has become more popular in recent years owing to their efficiency in the removal of pollutants too stable for biological methods. Dye adsorption is a result of two mechanisms (adsorption and ion exchange) and is influenced by many factors, such as dye/adsorbent interaction, adsorbent’s surface area, particle size, temperature, pH, and contact time. The main advantage of adsorption recently became the use of low-cost materials, which reduces the procedure cost.

**Figure 1:**
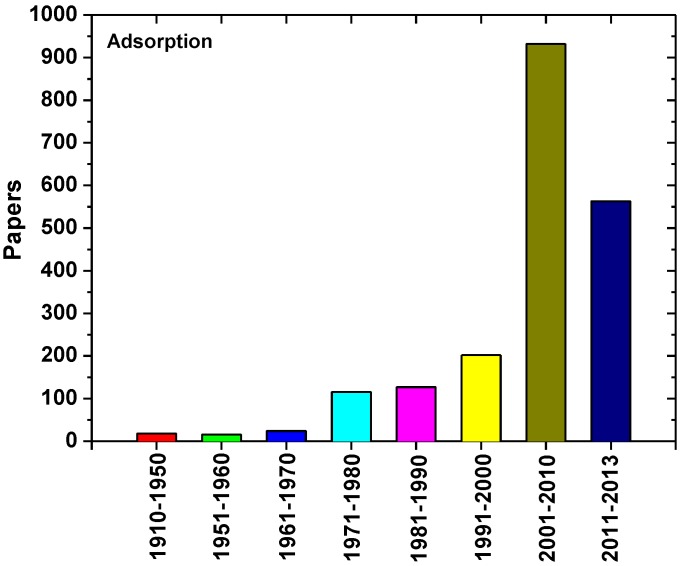
Works published for “dye adsorption” terms (Data after search in Scopus).

According to a brief screening in Scopus, numerous results were exported for the term “dye adsorption” (Figure 1). The major peak for this process was observed in 21st century. The present review firstly introduced the technology process, research history, and hotspot of adsorption. Their application in dyeing wastewater was then described in details. Major conclusions were exported for the differences of procedures during decades, which strongly influenced by new trends and economic aspects of each time-period. All of these confirmed the sustainability of adsorption techniques. Another point of interest is the different philosophy of adsorbent materials used during the last decades, becoming obvious, and the turn to low-cost materials.

## 2. Adsorbents for Dyeing Wastewaters

**Figure 2:**
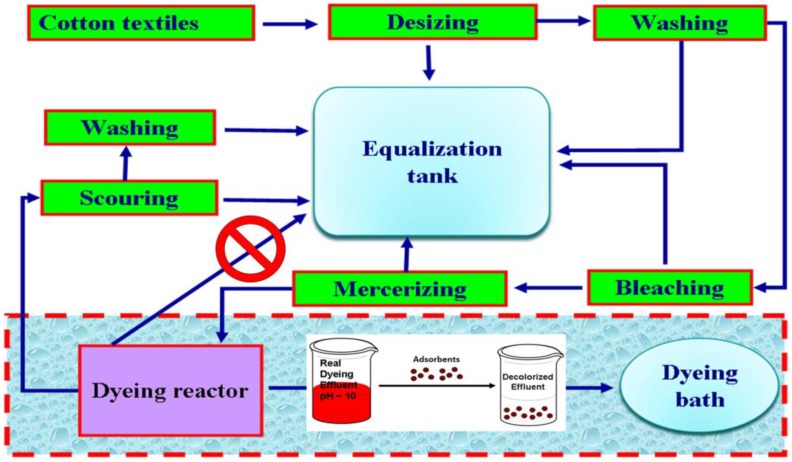
Treatment of textile wastewaters [30].

Adsorption has been applied either in a single mode, mainly for dyes removal from simulated/synthetic wastewaters, or in a combinational mode for total cleaning of real wastewaters. Recently, other materials, more economical, have been attempted to be used as adsorbents at the tertiary stage of effluent’s treatment, replacing the activated carbon: natural materials, biosorbents, waste materials from industry and agriculture, clay materials (bentonite, kaolinite), zeolites, siliceous material (silica beads, alunite, perlite), agricultural wastes (bagasse pith, maize cob, rice husk, coconut shell) [24,25,26], industrial waste products (waste carbon slurries, metal hydroxide sludge, coffee wastes) [24,27,28,29], biosorbents (chitosan, peat, biomass), and others (starch, cyclodextrin, cotton) [24,30]. In particular, for the treatment of textile wastewater, the most promising proposed technique was to lead the effluents from dyeing reactor to adsorption columns instead of an equalization tank [30] (Figure 2).

## 3. Change from Past to Future

### 3.1. First Attempts (1910–1950)

A first published paper for dye adsorption was in 1912, when Chapman and Siebold [31] attempted to separate particular dye molecules with adsorption techniques. However, that study was more in the analytical (and not technological) field given the limited knowledge. Furthermore, dyes were removed from solutions with growing crystal of sodium nitrate as materials, lead nitrate and barium nitrate in continuous 10-year studies [32,33]. A more particular study investigated the removal of wool violet 4BN from lead sulfate, giving acidity (pH) optimum conditions [34]. Based on previous knowledge, Gibby and Argument [35] studied the adsorption/interactions of Methylene Blue, Congo Red, Bordeaux Extra, Indigo Carmine, and Solway Ultra Blue using, as adsorbent materials, some mercury interfaces. It presented that the adsorption passed through a maximum as the concentration increased and the adsorption was calculated with Gibbs’s equation.

### 3.2. Initial Knowledge (1951–1970)

Based on the knowledge from previous years for dye adsorption, Ewing and Liu [36] studied the adsorption of crystal violet and Orange II from aqueous solutions on anatase, rutile, and zinc oxide. The nature of the adsorbent materials used began to differ and promoted some oxides. It was found that up to nine days of agitation were required to attain true equilibrium, and that high temperatures hastened the process. The adsorption has been found to be physical in nature. Whetstone [37] studied the importance of dye adsorption in crystal habit modification phenomena. Studies of modified crystals were realized, in which dye adsorption followed by overgrowth of dye molecules. Haldeman and Emmett [38], investigated the specific adsorption of the alkyl orange dyes on silica gels prepared in the presence of those dyes. The results confirmed that silica gel samples had a specific adsorptive capacity for the particular alkyl orange present during the gel preparation. The specificity gradually disappeared when the gels are stored at room temperature. Prasad and Dey [39] made a more thermodynamic study for dye adsorption, calculating the heats of adsorption of Congo Red and Fuchsine by using various samples of hydrous thorium oxide. The order of the heats of adsorption was in conformity with the order of the specific adsorption of the dyes by the samples.

The change in studies from the initial years of dye adsorption research to the future would be more intensely indicated by Brooks [40], who explained the mechanism of Methylene Blue adsorption from aqueous solutions, in quite different adsorbent systems, as common siliceous minerals found in petroleum reservoir formations. Three minerals (montmorillonite, kaolinite, and silica-sand flour) were prepared so that all the cec (cation exchange capacities) sites on the mineral surfaces were occupied by sodium. Methylene Blue dye adsorption isotherms were measured on these three minerals prepared in the sodium form. Other studies [41,42] showed she isotherms of some cyanine dyes adsorbed on surfaces of silver-halide precipitates. The specific areas of the precipitates were derived from measurements of the adsorption of argon and of benzene vapor. It was found that a given cyanine dye did not necessarily adsorb with the same area per molecule for conditions of saturation on each of the surfaces of silver chloride, bromide, and iodide. A first approach to porous and ceramic adsorbent materials was attempted in 1969 by Davis *et al.* [43], who described the dye adsorption on the surfaces of untreated and pretreated ceramic raw materials. These ceramic materials have not been used before, so the challenge would be very interesting. The adsorption of dye on alumina, bone, ceramic colors, and flint could be related to the specific surface areas. Porous materials such as flint exhibit a hysteresis effect, which was manifestly a difference in the rates of adsorption and of desorption.

### 3.3. Economic Development (1971–2000)

During this period, many changes were realized regarding the adsorption of model dyes from aqueous solutions. New materials, new parameters, and, in general, a novel philosophy of adsorption were developed. Iyer *et al.* [44] presented results of an experimental investigation in which effects of electrolytes in the aqueous dyebath on the various surfaces were studied. In experiments, the anionic direct dye Chlorazol Sky Blue FF was used for dyeing samples of viscose rayon, amorphous titanium dioxide, silica (aerosil), graphene, and activated charcoal. Adsorption thermodynamic parameters of samples tested were determined as function of electrolyte concentration. A first approach on dyeing wastewaters was done by Rock and Stevens [45], studying a combined process involving adsorption on synthetic polymer and ion exchange. The process had several operating and performance advantages over activated carbon adsorption. A more complete works was realized in 1975 by Sethuraman and Raymahashay [46], performing experiments to study the kinetics of adsorption of two industrial dyes [Methylene Blue (cationic) and Sulfur Blue (anionic)] by kaolinite and montmorillonite clays. The cationic dye was removed from aqueous solution at a continuously decreasing rate from 10 to 0.07 mg·g^−1^ min^−1^ by montmorillonite whereas kaolinite adsorbed the dye at a faster and uniform rate of 16 mg·g^−1^·min^−1^. The anionic dye was removed at a uniform rate of 2.3 mg·g^−1^·min^−1^ by kaolinite and 2.6 mg·g^−1^·min^−1^ by montmorillonite.

Given the economic boom of this period, other parameters began to be taken into consideration apart from the clearly scientific ones (pH, kinetics, capacity *etc.*). Therefore, Mitchell *et al.* [47] investigated the effectiveness of dye removal from textile wastewaters using low-cost activated carbons produced from various solid wastes. A first turn to economic low-cost materials was attempted. In some instances, activated carbons produced from solid wastes could be effectively used to remove residual dye from waste water. The effectiveness of dye removal by activated carbons produced from solid wastes varied from excellent, with carbon produced from peanut hulls, to poor, with carbon produced from pine bark. Basic and direct dye solutions were reduced to less than 1 ppm concentration using activated carbon produced from peanut hulls. Until then, activated carbon adsorption appeared to be the most effective and economical tertiary treatment method for removal of organics, including dyes. Potential sources of low cost activated carbon were agricultural, industrial, and municipal solid wastes. Further work of real industrial dyeing wastewaters was done by McKay [48], who studied the ability of Filtrasorb 400 activated carbon to absorb Astrazone Blue (basic) and Telon Blue (acidic) dyes using batch equilibrium and fluidized beds. The carbon adsorbed between 30% and 80% of its own weight of dye at equilibrium depending on dyestuff type and adsorbent particle size. The effects of adsorbent particle size and dye flow rates were also studied in fluidized bed systems. A detailed study of the adsorption of Telon Blue dye on carbon has been also undertaken using fixed beds, and the data correlated into a design model using the bed depth service time (BDST) method of analysis [49]. In this time-period, emphasis was given by many researchers to the study of diffusion phenomena of dye molecules onto adsorbents during procedure [50,51,52,53].

### 3.4. 21st Century

#### 3.4.1. Activated Carbon

Amongst all the adsorbent materials proposed in 21st century, activated carbon is the most popular for the removal of pollutants from wastewater [54,55]. In particular, the effectiveness of adsorption on commercial activated carbon for removal of a wide variety of dyes from wastewaters has made it an ideal alternative to other expensive treatment options [55]. Due to their great capacity to adsorb dyes, activated carbons are considered to be the most effective adsorbent. This capacity is mainly due to their structural characteristics and their porous texture, which gives them a large surface area, and their chemical nature, which can be easily modified by chemical treatment in order to increase their properties. Some of the main examples of wastewaters decolorization with activated carbon are given below.

The adsorption capacity of activated carbon depends on various factors, such as surface area, pore size distribution, and surface functional groups on the adsorbent, polarity, solubility, and molecular size of the adsorbate, solution pH and the presence of other ions in solution, and so on. The most widely used activated carbons are microporous and have high surface areas, and as a consequence, show high efficiency for the adsorption of low molecular weight compounds and for larger molecules. An adsorption study of Methylene Blue (MB) on activated carbon fiber (ACF) was also carried out. It has been used in adsorption systems, including removal of noxious gases, due to its extensive specific surface area, high adsorption capacity, well-developed micropores, reproducibility, and processability. The effects of various experimental parameters, such as the initial MB concentration and the ACF mass, on the adsorption rates were investigated. Equilibrium data was fit well by a Freundlich isotherm equation. Adsorption measurement shows that the process is very fast [56]. Nakagawa *et al.* [57] made attempt to evaluate the porous properties and hydrophobicity of activated carbons obtained from several solid wastes, namely, waste PET (polyethylene terephthalate), waste tires, refuse derived fuel, and wastes generated during lactic acid fermentation from garbage. Activated carbons having various pore size distributions were obtained by the conventional steam—activation method and via the pretreatment method (*i.e.*, mixture of raw materials with a metal salt, carbonization, and acid treatment prior to steam activation). The liquid-phase adsorption characteristics of organic compounds from aqueous solution on the activated carbons were determined to confirm the applicability of these carbons, where reactive dye (Reactive Black 5) was employed as representative adsorbate. Authors reported that the activated carbons with plentiful mesopores, prepared from PET and waste tires, had quite a high adsorption capacity for large molecules. Therefore, they are useful for wastewater treatment, especially for removal of bulky adsorbates. Li *et al.* [58] reported the displacement of atrazine by the strongly competing fraction of natural organic matter (NOM) in batch and continuous-flow powdered activated carbon (PAC) adsorption systems. The extent of atrazine displacement by NOM was found to be dependent on the type of PAC, while the rate of displacement was a function of PAC type as well as carbon dose. Choy *et al.* [59] reported the adsorption of three acidic dyes, Acid Blue 80 (AB 80), Acid Red 114 (AR 114), and Acid Yellow 117 (AY 117) onto activated carbon. In the same paper, they have also reported the adsorption isotherms for the three single components (AB 80, AR 114, AY 117) and three binary components (AB 80 + AR 114, AB 80 + AY 117, AR 114 + AY 117), dyes’ adsorption on activated carbon. Four models for predicting the multi—component equilibrium sorption isotherms have been compared in order to determine the best to predict or correlate binary adsorption data. These four models are the extended Langmuir isotherm, the simplified model based on single-component equilibrium factors, a modified extended Langmuir isotherm with a constant interaction factor, and a modified extended Langmuir isotherm incorporating a surface coverage—dependent interaction factor. Adsorption of trichloroethylene (TCE) by two ACFs and two granular activated carbons pre-loaded with hydrophobic and transphilic fractions of NOM were examined by Karanfil *et al.* [60]. In addition, the kinetics and mechanism of MB adsorption on commercially activated carbon and indigenously prepared activated carbons from bamboo dust, coconut shell, groundnut shell, rice husk, and straw have been reported by Kannan *et al.* [61]. The contributions of Jirankova *et al.* [62] deal with study of the combined adsorption—membrane process for organic dye removal. Adsorption equilibrium and kinetics of Egacid red sorption on PAC were studied in batch experiments. Tapered bed adsorption columns, using activated carbon, have been used to study the removal of two organic pollutants, an acid dye, and parachlorophenol, from aqueous effluent by McKay *et al.* [63]. Equilibrium sorption isotherms were measured to provide the saturation capacity (Q_e_) of each pollutant by Chemviron Filtrasorb 400 carbon, for operating continuous adsorption columns. The Redlich–Peterson (R–P) isotherm gives the best-fit model to describe the sorption process of these organic pollutants. The conventional bed depth service time (BDST) model has not been applied to tapered beds before, as the linear velocity of fluid is continually changing along the column. Several others authors [64,65] have also tested activated carbon for the adsorption of various dyes. Pereira *et al.* [66] reported that the surface chemistry of a commercially activated carbon has been selectively modified, without changing significantly its textural properties, by means of chemical treatments, using HNO_3_, H_2_O_2_, NH_3_, and thermal treatments under a flow of H_2_ or N_2_, and they found that the surface chemistry of the activated carbon plays a key role in dye adsorption performance. For cationic dyes (basic), the acid oxygen-containing surface groups show a positive effect but thermally treated samples still present good performances, showing the existence of two parallel adsorption mechanisms involving electrostatic and dispersive interactions. The conclusions obtained for each dye individually were confirmed in the color removal from a real textile process effluent.

Another source of activated carbon is agricultural waste. A very detailed review of Demirbas [67] and Sharma [68] reported some plentiful agricultural waste and various unused plant parts which offered an inexpensive and renewable additional source of activated carbon (AC). These waste materials have little or no economic value and often pose great disposal problems. Thus, these waste materials are used in treated and untreated forms for the removal of dyes. A wide variety of activated carbon prepared from agro-waste such as pine wood, corn cob, fruit stones, nut shells, cassava peel, tapioca peel, bamboo, bagasse, rice husk, bark, leaves, and used tea leaves are described by Sharma [68].

Application of activated carbon prepared from rice husk, was estimated as a potential adsorbent for the removal of Malachite Green and found to have good adsorption capacity comparative to the activated carbon prepared from banana peel [69], date pits [70], rice husk [61], wood saw dust [71], orange peel [69], and sugarcane dust [72]. Garg *et al.* [73] demonstrated that the adsorption efficiency of sulphuric acid treated saw dust was higher than formaldehyde treated saw dust for the removal of malachite green. It was concluded that ACR (activated carbon) adsorption efficiency was unaffected by pH, while pH = 6–9 was optimum for dye removal by SDC (sulphuric acid treated sawdust carbon) and SD (sawdust). The adsorption of ash prepared from rice husk was found to be an effective adsorbent because of its high surface area and the volume. The maximum monolayer capacity estimated was 690 mg/g. Moreover the adsorption was maximum on the ash compared to the activated carbon prepared from the rice husk after calcinations because of the presence of both silica and carbon. The adsorption characteristics of Direct Red 23 (DR 23) on to mangrove bark (Rhizopus apiculata), that has been previously treated with formaldehyde in acid medium, was investigated and observed that dye sorption decreases at high pH values in accordance with the ion exchange mechanism of the adsorption and maximum removal was at 2. The monolayer sorption capacity of modified bark for Direct Red 3 (DR 3) sorption was found to be 21.55 mg/g.

However, activated carbon presents several disadvantages [27]. It is quite expensive, the higher the quality the greater the cost, non-selective, and ineffective against disperse and vat dyes. The regeneration of saturated carbon is also expensive, not straightforward, and results in loss of the adsorbent. The reactivation or the regeneration of activated carbons involves restoring the adsorptive capacity of saturated activated carbon by desorbing adsorbed contaminants on the activated carbon surface.

The most common regeneration technique employed in industrial processes is thermal reactivation [74]. The thermal regeneration process generally follows three steps [75]: (i) adsorbent drying at approximately 105 °C; (ii) high temperature desorption and decomposition (500–900 °C) under an inert atmosphere; and (iii) residual organic gasification by an oxidizing gas (steam or carbon dioxide) at elevated temperatures (800 °C).

The heat treatment stage utilizes the exothermic nature of adsorption and results in desorption, partial cracking and polymerization of the adsorbed organics. The final step aims to remove charred organic residue formed in the porous structure in the previous stage and re-expose the porous carbon structure, regenerating its original surface characteristics. After treatment, the adsorption column can be reused. Per adsorption-thermal regeneration cycle between 5 and 15 wt % of the carbon bed is burnt off, resulting in a loss of adsorptive capacity [76]. Thermal regeneration is a high energy process due to the high required temperatures, making it both an energetically and commercially expensive process [75]. Plants that rely on thermal regeneration of activated carbon have to be of a certain size before it is economically viable to have regeneration facilities onsite. As a result, it is common for smaller waste treatment sites to ship their activated carbon cores to a specialized facility for regeneration, increasing the process' already significant carbon footprint [77].

Current concerns with the high energy/cost nature of thermal regeneration of activated carbon have encouraged research into alternative regeneration methods to reduce the environmental impact of such processes. Though several of the regeneration techniques cited have remained areas of purely academic research, some alternatives to thermal regeneration systems have been employed in industry. Current alternative regeneration methods are: (i) chemical and solvent regeneration [78]; (ii) microbial regeneration [79]; (iii) electrochemical regeneration [80]; ultrasonic regeneration [81]; and (iv) wet air oxidation [82].

#### 3.4.2. Chitosan-Based Adsorbents

There is abundant literature concerning the evaluation of adsorption performances of raw chitosan, especially in terms of adsorption capacity (amount of dye adsorbed) or uptake. In a batch system, the determination of the dye uptake rate by a chitosan-based material is often based on the equilibrium state of the adsorption system. At least 100 dyes, mainly anionic dyes, have been so far studied. Chitosan has an extremely high affinity for many classes of dyes. In particular, it has demonstrated outstanding removal capacities for anionic dyes such as acid, reactive, and direct dyes. This is due to its unique polycationic structure. The effectiveness of chitosan for its ability to interact with dyes has been studied by numerous workers. Juang and co-workers [83,84,85] demonstrated the usefulness of chitosan for the removal of reactive dyes. They found that the maximum adsorption capacities of chitosan for RR 222 (reactive red), RB 222 (reactive blue), and RY 145 (reactive yellow) were 1653, 1009, and 885 mg/g, respectively [84]. Annadurai [86,87,88] and Crini *et al.* [89] also reported that chitosan may be a useful adsorbent for the effluent of textile mills because of its high adsorption capacity. Uzun and Guzel [90,91,92,93] noted that chitosan can be used in the study of dyestuff adsorption in comparison with most other adsorbents. This polysaccharide showed a higher capacity for adsorption of dyes than CAC (commercial activated carbon) and other low-cost adsorbents, as reviewed by Crini [94]. Kim and Cho [95] also indicated that the amount of RB 5 adsorbed on chitosan beads is much greater than on CAC. Similar conclusions were reached by Lima *et al.* [96] for BB 9 (basic blue) adsorption. McKay’s group [97,98,99] recently published a series of papers on the ability of chitosan to act as an effective adsorbent for the removal of acid dyestuffs from aqueous solution. The monolayer adsorption (saturation) capacities were determined to be 973.3, 922.9, 728.2, and 693.2 mg of dye per gram of chitosan for AO 12 (acid orange), AO 10 (acid orange), AR 73 (acid red), and AR 18 (acid red), respectively [97]. The interaction between chitosan and anionic dyes has also been intensively investigated by Guibal and co-workers [100,101,102,103]. Their investigations clearly indicated that chitosan had a natural selectivity for dye molecules and was very useful for the treatment of wastewater. They reported that adsorption capacities ranged between 200 and 2000 mmol/g for chitosan and between 50 and 900 mmol/g for CAC [102]. They concluded that chitosan exhibited a twofold, or more, increase in the adsorption capacity compared to CAC in the case of acid, direct, reactive, and mordant dyes. The best choice for the adsorbent between CAC and chitosan depends on the dye, however, it was impossible to determine a correlation between the chemical structure of the dye and its affinity for either carbon or chitosan.

However, a strong advantage of chitosan is its modification ability. The basic idea of modifications is to make various changes in chitosan structure to enhance its properties (capacity, resistance, *etc.*). In particular, several researchers have proposed certain modifications in chitosan backbone to improve its adsorption capacity. These modifications are realized with grafting reactions [89,104]. The modifications can improve chitosan’s removal performance and selectivity for dyes, alter the physical and mechanical properties of the polymer, control its diffusion properties, and decrease the sensitivity of adsorption to environmental conditions. Many scientists suggested chemical grafting specific ligands [105,106]. However the only class for which chitosan [107] has low affinity is basic (cationic) dyes. To overcome this problem, the use of *N*-benzyl mono and disulfonate derivatives of chitosan is suggested to enhance its cationic dye hydrophobic adsorbent properties and to improve its selectivity [89,104]. These derivatives could be used as hydrophobic adsorbents in acidic media without any cross-linking reactions. To enhance and further develop the high potentials of chitosan, it is necessary to add/introduce chemical substituents at a specific position in a controlled manner [107]. This chemical derivatization promotes new adsorption properties, in particular towards basic dyes in acidic medium or reactive/acid dyes in basic medium. Another study deals with the enzymatic grafting of carboxyl groups onto chitosan as a mean to confer the ability to adsorb basic dyes on beads [107]. The presence of new functional groups on the surface of beads results in increased surface polarity and density of adsorption sites and hence improved adsorption selectivity for the target dye. Other studies showed that the ability of chitosan to selectively adsorb dyes could be further improved by chemical derivatization. Novel chitosan-based materials with long aliphatic chains are developed by reacting chitosan with high fatty acids and glycidyl moieties [108]. In this way, these products could be used as effective adsorption materials for both anionic and cationic dyes. Other researchers suggested the use of cyclodextrin—grafted chitosan derivatives as new chitosan derivatives for the removal of dyes [109,110]. These materials are characterized by a rate of adsorption and a global efficiency greater than that of the parent chitosan polymer [107].

The pure form of chitosan powders (raw) tends to present some disadvantages, such as unsatisfactory mechanical properties and poor heat resistance. Another important limitation of the pure form is its solubility in acidic media and, therefore, it cannot be used as an insoluble adsorbent under these conditions (except after physical and chemical modification). The main technique to overcome these limitations is to transform the raw polymer into a form in which physical characteristics are more attractive. So, cross-linked beads have been developed and proposed. After cross-linking, these materials maintain their properties and original characteristics [111], particularly their high adsorption capacity, although this chemical modification results in a decrease in the density of free amine groups at the surface of the adsorbent in turn lowering polymer reactivity towards metal ions [100,101]. The cross-linking agent is very important. Therefore many researchers studied the chitosan behavior prepared with different cross-linkers, such as glutaraldehyde (GLA), tripolyphosphate sodium (TPP), epichlorydrine (EPI), ethylene glycol diglycidyl ether (EGDE), *etc.* [18,20,112,113]. The change in adsorption capacity was confirmed; the results showed that the chitosan—EPI beads presented a higher adsorption capacity than GLA and EGDE [20,113]. They reported that these materials can be used for the removal of reactive, direct, and acid dyes. It was found that 1 g chitosan adsorbed 2498, 2422, 2383, and 1954 mg of various reactive dyes (Reactive Blue 2; Reactive Red 2; Direct Red 81; and Acid Orange 12, respectively) [18]. As a comparison, it is specified that the adsorption capacities of commercially activated carbon for reactive dyes generally vary from 280 to 720 mg/g. Another advantage of EPI is that it does not eliminate the cationic amine function of the polymer, which is the major adsorption site to attract the anionic dyes during adsorption [113]. The cross-linking with GLA (formation of imine functions) or EDGE decreases the availability of amine functions for the complexation of dyes. With a high cross-linking ratio the uptake capacity decreases drastically. Among the conditions of the cross-linking reaction that have a great impact on dye adsorption is the chemical nature of the cross-linker, as mentioned above, and also the extent of the reaction. In general, the adsorption capacity depends on the extent of cross-linking and decreases with an increase in cross-linking density. When chitosan beads were cross-linked with GLA under heterogeneous conditions, it was found that the saturation adsorption capacity of reactive dyes on cross-linked chitosan decreased exponentially from 200 to 50 mg/g as the extent of cross-linking increased from 0 to 1.5 mol GLA per mol of amine. This is due to the restricted diffusion of molecules through the polymer network and reduced polymer chain flexibility. In addition, the loss of amino-binding sites by reaction with aldehyde is another major factor in this decrease. However, the cross-linking step was necessary to improve mechanical resistance, to enhance the resistance of material against acid, alkali, and chemicals, and also to increase the adsorption abilities of chitosan. According to literature [18,20,112,113], the adsorption capacity of non cross-linked beads was greater than that of cross-linked beads in the same experimental conditions. The materials, mainly cross-linked using GLA, have been also proposed as effective dye adsorbents by several researchers [109,111]. The reaction of chitosan with GLA leads to the formation of imine groups, in turn leading to a decrease in the number of amine groups, resulting in a lowered adsorption capacity, especially for dyes adsorbed through ion-exchange mechanisms. Furthermore, it is noted that cross-linking can change the crystalline nature of chitosan, as suggested by the X-ray diffraction (XRD) diffractograms. After the cross-linking reaction, there was a small increase in the crytallinity of the chitosan beads and also increased accessibility to the small pores of the material.

It is evident from this brief literature survey that chitosan can be utilized as an interesting tool for the purification of dye-containing wastewater because of its outstanding adsorption capacity.

#### 3.4.3. Low-Cost Agricultural Wastes as Adsorbents

There have been many attempts to find inexpensive and easily available adsorbents to remove pollutants such as agricultural solid wastes where, according to their physicochemical characteristics and low cost, they may be good potential adsorbents [114]. Agricultural productions are available in large quantities around the world; thus large amounts of waste are rejected. Table 1 shows agricultural production (ton/year) in some countries. Agricultural wastes are lignocellulosic materials that consist of three main structural components, which are lignin, cellulose, and hemicelluloses. These components contribute mass and have high molecular weights. Lignocellulosic materials also contain extractive structural components, which have a smaller molecular size [67]. Agricultural wastes are renewable, available in large amounts and are less expensive as compared to other materials used as adsorbents. Agricultural wastes are better than other adsorbents because agricultural waste is usually used without or with a minimum of processing (washing, drying, grinding) and, thus, reduce production costs by using a cheap raw material and eliminating energy costs associated with thermal treatment [115]. There are specific alternative agricultural by-products used intensely as dye adsorbents, such as peanut hull, coir pith, and rice husk, as listed in Table 1, which shows previous studies of the adsorption of different dyes using adsorbents based on agricultural solid wastes.

**Table 1:** Previous studies of the adsorption of dyes using adsorbents based on agricultural solid wastes.

Adsorbent	Dye	Reference
Sugar beet pulp	Gemazol turquoise blue-G	[116]
Powdered peanut hull	Sunset yellow, Amaranth, Fast green	[117]
Rice husk ash	Indigo Carmine	[118]
Chemically modified peanut hull	Methylene Blue, Brilliant cresyl blue, Neutral red, Sunset yellow, Fast green	[119]
Peanut hull	Methylene Blue, Brilliant cresyl blue, Neutral red	[120]
Coir pith activated carbon	Reactive Orange 12, Reactive Red 2, Reactive Blue 4	[121]
Coir pith activated carbon	Congo Red	[3]
Coir pith carbon	Methylene Blue	[122]
ZnCl_2_ activated coir pith carbon	Acid brilliant blue, Acid violet, Methylene Blue, Rhodamine B	[123]
Coir pith	Acid violet	[71]
Rice husks activated carbon	Malachite green	[124]
Rice husk-based porous carbon	Malachite green	[125]
Rice husk	Congo Red	[126]
Tea waste	Methylene Blue	[127]
Coniferous pinus bark powder	Crystal violet	[128]
Orange peel activated carbon	Direct Blue-106	[129]
Neem sawdust	Malachite green	[130]
Guava seed carbon	Acid blue 80	[131]
Peanut hull	Reactive Black 5	[132]
Loofa activated carbon	Reactive orange	[133]
Apricot stone activated carbon	Astrazon yellow 7 GL	[134]
Almond shells	Direct red 80	[135]
Lemon peel	Malachite green	[136]
Bagasse fly ash	Methyl violet	[137]
Polygonum orientale Linn activated carbon	Malachite green	[138]

#### 3.4.4. Fungi, Bacteria, Algae

Three novel-trendy categories of recent literature for the decolorization of dyeing wastewaters with adsorption are fungi, bacteria, and algae [139].

A wide variety of fungal organisms are capable of decolorizing a wide range of dyes [140,141]. Many genera of fungi have been employed either in living or inactivated form. The use of white-rot fungi such as *Phanerochaete chrysosporium* in decolorizing textile wastewater has been widely reported in literature [142,143,144,145,146,147,148,149]. There are various fungi other than white-rot fungi, such as *Aspergillus niger* [141,150,151], *Rhizopus arrhizus* [152], *Rhizopus oryzae* [153], which can also decolorize and/or biosorb diverse dyes. For living cells, the major mechanism is biodegradation because they can produce lignin modifying enzymes, laccase, manganese peroxidase (MnP), and lignin peroxidase (LiP), which due to their unspecific activity are the first step in the process of mineralization of dyes [154]. The relative contributions of LiP, MnP, and laccase to the decolorization of dyes may be different for each fungus. In addition to biodegradation, a biosorption mechanism might also play an important role in the decolorization of dyes by living fungi. For dead cells, the mechanism is biosorption, which involves physicochemical interactions such as adsorption, deposition, and ion exchange. Decolorization of dye wastewater by fungal biomass has been extensively reviewed by Fu and Viraraghavan [140], Kaushik and Malik [155], and Singh [156]. Limited information is available on interactions between dead fungal biomass and a variety of dyes with complex molecular structures. Fu and Viraraghavan [157] studied the roles played by functional groups such as carboxyl, amino, phosphate, and lipid fractions present in fungal biomass from *Aspergillus niger* in the biosorption of four different dyes. In the biosorption of Basic Blue 9 on *Aspergillus niger*, carboxyl and amino groups were found to be the main binding sites while in biosorption of Acid Blue 29, only the amino group was a major site, and electrostatic attraction was believed to be the primary mechanism. In the biosorption of Congo Red, the amino, carboxylic acid, phosphate groups, and lipid fractions were all found to be important binding sites and, in addition to electrostatic attraction, other mechanisms were also believed to be involved in biosorption. In the biosorption of Disperse Red 1, physical and chemical adsorption along with electrostatic attraction was found to be the mechanism of biosorption while amino group and lipid fractions were the major binding sites.

Most research has focused on studying the biodegradation/decolorization potential of bacteria [158] and less attention has been paid to employing dead bacterial biomass for biosorption of dyes. In 2005, Won *et al.* [159] identified *Corynebacterium glutamicum* as a potential biosorbent of Reactive Red 4, which can bind 104.6 mg/g at pH 1. Two general types of bacteria exist, Gram-positive and gram-negative. Gram-positive bacteria are comprised of a thick peptidoglycan layer connected [160,161] by amino acid bridges. Embedded in the Gram-positive cell wall are polyalcohols, some of which are lipid linked to form lipoteichoic acids. The cell wall of Gram-negative bacteria is thinner, and composed of only 10%–20% peptidoglycan [162]. In addition, the cell wall contains an additional outer membrane composed of phospholipids and lipopolysaccharides. The mode of solute uptake by dead/inactive cells is extracellular; the chemical functional groups of the cell wall play vital roles in biosorption. Biodegradation processes may be anaerobic, aerobic, or involve a combination of the two. The ability of actinomycetes, particularly Streptomyces species, to decolorize and degrade textile dyes has been widely investigated. In a study by Hu [163], three Gram-negative bacteria (*Aeromonas sp.*; *Pseudomonas luteola*; and *Escherichia coli*), two Gram-positive bacteria (*Bacillus subtilis* and *Staphylococcus aureus*) and activated sludge (consisting of both gram-negative and gram-positive bacteria) were used as biosorbents for the removal of reactive dyes such as Reactive Blue, Reactive Red, Reactive Violet, and Reactive Yellow. Dead cells of test genera showed a higher uptake than living cells due to an increased surface area and gram-negative bacteria had a higher adsorption capacity than gram-positive bacteria due to higher lipid contents in the cell wall portion. The mode of solute uptake by dead/inactive cells is extracellular, and the chemical functional groups of the cell wall play vital roles in biosorption. Functional groups present on the bacterial cell wall include carboxyl, phosphonate, amine and hydroxyl groups [164]. Several dye molecules, which exist as dye cations in solutions, are also attracted towards carboxyl and other negatively charged groups. In addition, amine groups adsorb anionic dyes via electrostatic interaction or hydrogen bonding. Vijayaraghavan and Yun [165] observed that the amine groups of *Corynebacterium glutamicum* were responsible for the binding of reactive dye anions via electrostatic attraction. Carboxyl, amine, phosphonate, sulfonate, and hydroxyl groups have become well established as being responsible for dye binding.

Algae have been found to be potential biosorbents because of their availability in both fresh and saltwater. The biosorption capacity of algae is attributed to their relatively high surface area and high binding affinity. Cell wall properties of algae play a major role in biosorption; electrostatic attraction and complexation are known to take place during algal biosorption [166]. Functional groups such as hydroxyl, carboxylate, amino, and phosphate, found on the algal cell surface, are considered to be responsible for sequestration of contaminants from wastewater. The dye removal, especially by using algae, may be attributed to the accumulation of dye ions on the surface of algal biopolymers and further to the diffusion of the dye molecules from the aqueous phase to the solid phase of the biopolymer [167]. Extracellular polymers consist of surface functional groups, which enhance sorption of the dye molecules onto the surface of the polymer (floc) during the dye removal process [168,169]. The released metabolic intermediates (long chain biopolymers), which have excellent coagulation capacity, along with the dye remaining in the aqueous phase, tend to adsorb onto the surface of the polymers and settle (biocoagulation) [169]. Mohan *et al.* [169] studied the removal of Reactive Yellow 22 dye by active *Spirogyra sp*. and reported that the dye removal mechanism can be explained by biosorption, bioconversion, and biocoagulation. While, removal of Acid Red 274 dye using an inactivated *Spirogyra rhizopus* system was attributed to biosorption and biocoagulation [167]. A study by Ozer *et al.* [167] revealed the potential ability of the algae *Spirogyra rhizopus* to decolorize synthetic wastewaters containing initial concentration of dye Acid Red 274 from 25 to 1000 mg/L. Almost complete removal of Acid Red 274 dye from synthetic wastewater, with a final concentration lower than 25 mg/L, was achieved by using *Spirogyra rhizopus* resulting from biocoagulation and biosorption.

#### 3.4.5. Nanomaterials

A relatively new category of adsorbents (especially regarding their size) is nanomaterials. After 1995, some attempts were achieved in order to prepare and use these nanosized materials to decolorize effluents or simply remove dyes from aqueous systems.

A raw attempt was done by Wu and co-workers [170], who investigated the adsorption of several anionic dyes on nanosized alumina-modified silica particles of different compositions. Those silica cores had the same surface properties as alumina dispersed in aqueous solutions. Khan *et al.* [171]. studied the synthesis of nanoscale zero valent iron (NZVI) and tested it for the purification of waste water contaminated by basic blue 3 dye. In other study [172], nanoporous silica (NPS), with an average pore diameter of 2.4 nm and a surface area of 949 m^2^/g, was synthesized by using nonyl phenol ethoxylated decylether (NP-10) and ethyl silicate 40% (ETS-40) under acidic conditions. Ionic liquid functionalized adsorbent was prepared by grafting of *N*-methyl-*N*′-propyltrimethoxysilylimidazolium chloride onto NPS for removal of methyl orange. Furthermore, Mahmodi used nickel ferrite nanoparticle (NFN) as dye nanomaterial [173]. The surface modification of NFN using sodium dodecyl sulfate (SDS) was studied. Dye removal ability of NFN and surface modified NFN (NFN-SDS) was investigated. Basic Blue 41 (BB 41), Basic Green 4 (BG 4), and Basic Red 18 (BR 18) were used as model compounds. The maximum dye adsorption capacity was 0.50 mg/g (BB 41), 0.41 mg/g (BG 4), and 0.25 mg/g (BR 18) for NFN and 111 mg/g (BB 41), 17 mg/g (BG 4), and 44 mg/g (BR 18) for NFN-SDS. Zhai *et al.* [174] reported the electrochemical synthesis of porous Pr(OH)_3_ nanobelt arrays (NBAs), nanowire arrays (NWAs), nanowire bundles (NWBs), and nanowires (NWs), and their applications as dye absorbents in water treatment. These Pr(OH)_3_ nanostructures exhibited high efficient and selective adsorption of the dyes with amine (-NH_2_) functional groups such as Congo Red, Reactive Yellow, and Reactive Blue.

Liu and Zhang [175] studied the adsorption properties of nanoclays (activated clays by acid-treatment or calcinations, organic-modified clays with small molecules or polymers) for the adsorption and removal of organic dyes from aqueous solutions. In general, clays are natural environment-friendly materials with high specific surface area and are now widely used for the adsorption and removal of the organic pollutants.

A study of Cheung *et al.* [176] was based on the preparation of nanochitosan emulsion in a suspension form by adding tripolyphosphate solution into a chitosan solution drop-wise. The adsorption capacities of four acid dyes, namely, Acid Orange 10 (AO 10), Acid Orange 12 (AO 12), Acid Red 18 (AR 18), and Acid Red 73 (AR 73) on nanochitosan, have been determined to be 1.77, 4.33, 1.37, and 2.13 mmol/L, respectively. The nanochitosan dye capacities were compared with normal chitosan capacities, which were 1.54, 2.66, 1.11, and 1.25 mmol/L for AO 10, AO 12, AR 18, and AR 73, respectively. In all cases, the nanochitosan has a higher capacity. A combination of chitosan and cyclodextrin was also probable as reported in other study [177]. Magnetic β-cyclodextrin-chitosan/graphene oxide materials (MCCG) were fabricated through a facile chemical route, and their application as excellent adsorbents for Methylene Blue (MB) removal were also demonstrated.

Another field of nanomaterials are the effects of magnetism. One of the first attempts to the concept of magnetic nano-dye-adsorbents was introduced by Liao *et al.* [178], who prepared them by covalently binding polyacrylic acid onto Fe_3_O_4_ magnetic nanoparticles, possessing high ion-exchange capacity and was successfully used for the fast removal of crystal violet. Another study reported the adsorption (and desorption) of Congo Red (CR) onto maghemite nanoparticles (γ-Fe_2_O_3_) [179]. The adsorption capacity was evaluated using both the Langmuir and Freundlich adsorption isotherm models. Maghemite nanoparticles (γ-Fe_2_O_3_) were prepared easily in a surfactant-less microemulsion by co-precipitation method. Deng *et al.* [180] studied the synthesis of magnetic graphene oxide (MGO) and its use as an adsorbent for simultaneous removal of Cd(II) and ionic dyes including Methylene Blue (MB) and Orange G (OG). In mono-component system, the maximum sorption capacities in ultrapure water for MB and OG were 64.23 and 20.85 mg/g, respectively. Furthermore, adsorption of seven different organic dyes from aqueous solutions onto magnetite nanoparticles loaded tea waste (MNLTW) was studied by Madrakian and co-workers [181]. MNLTW was prepared via a simple method and was fully characterized. Adsorption characteristics of the MNLTW adsorbent was examined using Janus Green, Methylene Blue, Thionine, Crystal Violet, Congo Red, Neutral Red, and Reactive Blue 19 as adsorbates. Dyes adsorption process was thoroughly studied from both kinetic and equilibrium points of view for all adsorbents. Ge *et al.* [182] explored the removal of cationic dyes (Crystal Violet, Methylene Blue, and Alkali Blue 6B) from water solution by adsorption with magnetic nanoparticles (MNPs) modified with 3-aminopropyltriethoxysilane and copolymers of acrylic acid and crotonic acid. In another study [183], a graphene-based magnetic nanocomposite was synthesized and used as an adsorbent for the removal of a dye (Fuschine) from aqueous solutions.

In addition, nanocarbons were recently reported as dye adsorbents. Gong and co-workers [184] dealt with magnetic multi-wall carbon nanotube (MMWCNT) nanocomposites. They were synthesized and used as adsorbents for removal of cationic dyes from aqueous solutions. The MMWCNT nanocomposite was composed of commercial multi-wall carbon nanotubes and iron oxide nanoparticles. Adsorption characteristics of the MMWCNT nanocomposite adsorbent were examined using Methylene Blue, neutral red and brilliant Cresyl Blue as adsorbates. Experiments were carried out to investigate adsorption kinetics, adsorption capacity of the adsorbent and the effect of adsorption dosage and solution pH values on the removal of cationic dyes. Mishra and co-workers [185] functionalized multiwalled carbon nanotubes (f-MWNTs) for the adsorption (decolorization) of three different azoic dyes. Multiwalled carbon nanotubes (MWNTs) were synthesized by chemical vapor deposition (CVD) technique and purified by air oxidation and acid treatment. Maximum adsorption capacity of 148, 152, and 141 mg/g was obtained for direct Congo Red, Reactive Green HE4BD, and Golden Yellow MR dyes, respectively.

Apart from the common nanoadsorbents, nanoporous agricultural wastes were also reported for decolorization. In a study by Jayarajan *et al.* [186], the effect of agricultural waste product nano-porous adsorbent of Jack fruit peel waste for removing dye Rhodamine dye (Rd) from aqueous solution was investigated. The effect of adsorption isotherm were studied by carrying out a series of isotherm at different adsorbent dosages (1, 2, and 3 g/L), temperatures (30, 40, and 60 °C) and pH (4.95, 8.14, and 9.74), respectively. The adsorption equilibrium data were analyzed by using various adsorption isotherm models and the results have shown that adsorption behavior of the dye could be described reasonably well by Langmuir and Freundlich models. The characteristic parameters for each isotherm have been determined. The monolayer adsorption capacity determined was reasonably high (g/L) at adsorbent dosage 4.361 (g/L), temperature 2.8496 (g/L) and pH 4.3614 (g/L) for adsorption of Rd dye, respectively. The monolayer adsorption capacity was determined to be 4.361 to 1.98 mg/g.

### 3.5. Future Plans

The main advantage of adsorption technique is the opportunity to improve the nature and properties of adsorbent particles used. The trend of recent research leads to the application of cost-effective materials, which can adequately adsorb the dyes from solutions even presenting slightly lower capacities. The need of the market is to limit the procedure costs regarding the environmentally-friendly nature of the techniques suggested. For this purpose, significant emphasis will be given to green technology, using low-cost materials (agricultural wastes or by-products). There are already many works on this field, which are expected to be further improved.

The main drawback of the already published dye-adsorption studies is that their use is still in the laboratory stage, mostly without pilot studies or commercialization. Limited attempts for detailed economic and market analyses are available [139]. There is still reluctance in opting for non-viable immobilized biomass-based systems compared to the use of biological reactors with living cells, although the advantages of the former systems are well established. Some attempts have been realized in the past at commercializing immobilized biomass dye biosorbents such as Alga-Sorb, AMT-Bioclaim, B.V. Sorbex’s biosorbents, and Bio-fix, but none have made a successful commercial entry in the market [187,188,189]. Peat is considered as the most successful biosorbent for use in either a natural state or in a modified form; however it is not regarded as the best biomaterial for commercialization because it is a finite resource and it is also not available everywhere in the world [188].

The main future target of market is to move the dye adsorption process to an industrial scale. It is relatively less difficult to demonstrate it in a laboratory; it is a little more challenging to demonstrate it at a pilot scale, but to really increase it to a large scale would call for a significant financial and technological effort. This mismatch between scientific progress in biosorption research (biosciences) and stagnation in industrial technology innovation needs to be corrected through translational research and technology transfer with a push for commercialization of research. Universities can play an active role in this process through a more formalized approach to technology transfer and protection of intellectual property [139,190].

## 4. Conclusions

Adsorption as technique for dye adsorption was traced back in 1912. Numerous combinations between adsorbents and dye molecules were tested in order to obtain an effective material for industrial applications. From the past to the future, the nature of adsorbents used was significantly changed. From some inorganic hydroxides and crystals of 1950s, we passed to complex polymeric materials and carbons in 1990s, and now a trend to low-cost agricultural-based adsorbents. The key-factor of each dye adsorbent of the past was only adsorption capacity, but now many factors were introduced in order to study an effective dye adsorbent (pH, kinetic rate, ionic strength, adsorbent’s dosage, *etc.*). More attention was also given on the nature and especially porosity of adsorbents, creating a new promising class of nanomaterials. However, apart from all of the above, the main drawback of the already published dye-adsorption studies is that their use is still in the laboratory stage mostly without pilot studies or commercialization. Limited attempts for detailed economic and market analyses are available. The main future target of market is to move the dye adsorption process to an industrial scale. It is relatively less difficult to demonstrate it in a laboratory; it is a little more challenging to demonstrate it at a pilot scale, but to really increase it to a large scale would call for a significant financial and technological effort.
